# Cognitive performance in ISS astronauts on 6-month low earth orbit missions

**DOI:** 10.3389/fphys.2024.1451269

**Published:** 2024-11-20

**Authors:** Sheena I. Dev, Alaa M. Khader, Sydney R. Begerowski, Steven R. Anderson, Gilles Clément, Suzanne T. Bell

**Affiliations:** ^1^ NASA Behavioral Health and Performance Laboratory, KBR, Inc., Houston, TX, United States; ^2^ NASA Behavioral Health and Performance Laboratory, JES Tech, Houston, TX, United States; ^3^ NASA Behavioral Health and Performance Laboratory, NASA Johnson Space Center, Houston, TX, United States

**Keywords:** Cognitive performance, astronauts, low earth orbit, spaceflight, cognition, ISS

## Abstract

**Introduction:**

Current and future astronauts will endure prolonged exposure to spaceflight hazards and environmental stressors that could compromise cognitive functioning, yet cognitive performance in current missions to the International Space Station remains critically under-characterized. We systematically assessed cognitive performance across 10 cognitive domains in astronauts on 6-month missions to the ISS.

**Methods:**

Twenty-five professional astronauts were administered the Cognition Battery as part of National Aeronautics and Space Administration (NASA) Human Research Program Standard Measures Cross-Cutting Project. Cognitive performance data were collected at five mission phases: pre-flight, early flight, late flight, early post-flight, and late post-flight. We calculated speed and accuracy scores, corrected for practice effects, and derived z-scores to represent deviations in cognitive performance across mission phases from the sample’s mean baseline (i.e., pre-flight) performance. Linear mixed models with random subject intercepts and pairwise comparisons examined the relationships between mission phase and cognitive performance.

**Results:**

Cognitive performance was generally stable over time with some differences observed across mission phases for specific subtests. There was slowed performance observed in early flight on tasks of processing speed, visual working memory, and sustained attention. We observed a decrease in risk-taking propensity during late flight and post-flight mission phases. Beyond examining group differences, we inspected scores that represented a significant shift from the sample’s mean baseline score, revealing that 11.8% of all flight and post-flight scores were at or below 1.5 standard deviations below the sample’s baseline mean. Finally, exploratory analyses yielded no clear pattern of associations between cognitive performance and either sleep or ratings of alertness.

**Conclusion:**

There was no evidence for a systematic decline in cognitive performance for astronauts on a 6-month missions to the ISS. Some differences were observed for specific subtests at specific mission phases, suggesting that processing speed, visual working memory, sustained attention, and risk-taking propensity may be the cognitive domains most susceptible to change in Low Earth Orbit for high performing, professional astronauts. We provide descriptive statistics of pre-flight cognitive performance from 25 astronauts, the largest published preliminary normative database of its kind to date, to help identify significant performance decrements in future samples.

## 1 Introduction

Astronauts on current and future spaceflight missions are required to execute complex tasks in which even minor errors could have devastating consequences. Intact cognitive functioning is critical to maintain exceptional performance standards during long duration spaceflight missions. However, astronauts are exposed to spaceflight environmental stressors that include spaceflight hazards (i.e., radiation, altered gravity, isolation and confinement, hostile/closed environment, and distance from Earth) as well as operational challenges (e.g., work overload, circadian shifts, communication delays) that could compromise cognitive functioning (Slack et al., 2016). Some of these challenges are experienced by astronauts living and working on the International Space Station (ISS), yet the extent to which cognitive performance decrements are observed on ISS missions remains critically under-characterized.

Anecdotal and self-report evidence suggest some astronauts experience subjective cognitive difficulties inflight (Stuster, 2010; Stuster, 2016; Schroeder and Tuttle, 1992; Clément et al., 2020), particularly for tasks requiring sustained attention and speed of processing. However, objective assessments of cognitive performance in spaceflight have yielded mixed findings (For review see Thoolen and Strangman (2023)). Early studies assessing dual tasking performance in short duration shuttle missions suggest that attention may be adversely impacted during tasks that include a primary sensorimotor component (Heuer et al., 2003; Manzey et al., 1995; Manzey et al., 2000). More recent studies examining cognitive performance during 6-month ISS missions have largely focused on specific cognitive domains. For example, Jones et al. (2022) recently reported that astronauts who obtained 5 h or less of sleep on the ISS performed worse on a task of sustained attention. Relative to pre-flight, slowed reaction time on a task of spatial working memory post-flight but not during flight (Tays et al., 2021), decrements in visuospatial line orientation inflight and early post-flight (Takács et al., 2021), and increased error on a dual tasking paradigm on landing day (Moore et al., 2019) have been reported. The post-flight decrements observed in these studies returned to pre-flight levels over 30 days follow up. In contrast, no differences were reported in spatial working memory accuracy, processing speed, cognitive-dual tasking, and visual memory (Burles and Iaria, 2023; Moore et al., 2019; Tays et al., 2021). This body of literature is limited by small samples, lack of measurement standardization across studies, limited sensitivity of measures to detect change in high performing populations, and inconsistent timepoints within mission (Strangman et al., 2014). Further, the scope of cognitive domains assessed is restricted and there is an overall paucity of data documenting other cognitive functions that may impact mission relevant tasks, such as memory, executive functions, and emotional processing. Nevertheless, current evidence suggests at most mild and reversible decrements in aspects of cognition among astronaut crew on ISS missions of 6-month duration or shorter.

Only one published study to date has comprehensively assessed a wide range of cognitive domains across multiple mission phases in spaceflight. The well-known NASA Twin study (Garrett-Bakelman et al., 2019) conducted cognitive testing using the Cognition Battery (Basner et al., 2015) in a pair of monozygotic twins, one of which spent approximately 1 year (340 days) on the ISS while the other remained on Earth. Early inflight improvements were documented in speed and accuracy, with the exception of accuracy decrements in specific domains of visual memory and abstract reasoning (>1 standard deviation [SD] decline). Relative to early flight, late flight performance was slower in emotion recognition (>1SD) and less accurate in abstract reasoning (>2 SD). However, the most notable decrements were observed in the post-flight mission phase across most cognitive domains that persisted up to 6 months. Though a single case study, findings underscore the relevance of assessing a breadth of cognitive domains in spaceflight, support previous studies indicating that cognitive performance may be differentially impacted at different mission phases, and suggest that longer duration missions may induce more persistent decrements.

Terrestrial analogs that simulate spaceflight conditions offer a unique opportunity to study singular or combined spaceflight hazards and stressors on relevant behavioral health and performance outcomes. Group level changes in cognitive performance were not present in several long duration European, Russian, and Antarctic analog missions (Lorenz et al., 1996; Gemignani et al., 2014; Mairesse et al., 2019; Connaboy et al., 2020). However, a subset of individuals in these settings appear to be more vulnerable to decrements in aspects of attention, memory, or visuospatial abilities, including those who report depressive symptoms (Premkumar et al., 2013), heightened stress and sleep loss (Basner et al., 2014), and greater hippocampal volume loss (Stahn et al., 2019). Indeed, sleep restriction protocols implemented in one- and 2-week isolation and confinement spaceflight analogs were associated with worse emotion recognition, slower and less accurate sustained attention, and slower cognitive and psychomotor processing speed (Nasrini et al., 2020). Other studies also have examined the effects of altered gravity on cognition. Parabolic flight studies assessing impacts of acute gravitational alterations have documented decrements on tasks of spatial cognition (Stahn et al., 2020) and attention (Friedl-Werner et al., 2021). Head Down Bed Rest (HDBR) studies, designed to simulate cephalic fluid shift in microgravity, report that prolonged HDBR can induce early but mild reductions in overall cognitive speed that remain consistent for the duration of the protocol (Basner et al., 2021a) and more specific decrements on tasks of executive functioning (Yuan et al., 2016), emotional processing (Basner et al., 2021a; Brauns et al., 2019; Benvenuti et al., 2013; Liu et al., 2012), and aspects of memory (Chen et al., 2013). Decrements resolve soon after returning to normal daily activities and were only exacerbated by a combined HDBR and CO_2_ protocol in isolated tasks of executive function and sustained attention (Basner et al., 2021b; Lee et al., 2019; Mahadevan et al., 2021). Thus, analog studies have identified mild, possibly reversible, decrements in cognitive performance across several domains in the context of spaceflight stressors. Even among those that did not report significant group level differences, observed individual variability in performance suggests that some individuals are more at risk for cognitive decrements than others.

Taken together, the extant literature documenting cognitive performance in spaceflight or spaceflight analogs suggests possible mild decrements in some, but not necessarily all, cognitive domains and that these decrements may be singularly or synergistically influenced by multiple spaceflight hazards (e.g., altered gravity) and stressors (e.g., sleep restriction). Given the dynamic nature of spaceflight missions and thus the potential for hazards and stressors to interact in varying degrees within a mission, it is also possible that there are specific mission phases in which performance is more vulnerable to decrements. However, we were unable to locate a published study that has described the neuropsychological profile expected in low earth orbit spaceflight by systematically assessing cognitive performance across a wide range of cognitive domains in a cohort of astronauts aboard the ISS for 6-month missions.

The primary aims of the current study were to 1) provide a descriptive summary of performance across several cognitive domains in a sample of professional astronauts on 6-month ISS missions, 2) characterize cognitive performance over time and between distinct mission phases of a 6-month ISS mission, and 3) examine the prevalence of low scores across subtests. While we generally expected stable cognitive performance across time, we predicted that performance would be most vulnerable during mission phases that require greater adaptation to and from spaceflight conditions. Further, processing speed, attention, and working memory are cognitive domains which are more susceptible to state-like alterations due to internal or external distractions (Eysenck and Calvo, 1992; Robert and Hockey, 1997). Thus, we hypothesized that 1) there would be mild decrements in early flight and early post-flight mission phases relative to pre-flight; and 2) the subtests with the greatest frequency of low scores would be those assessing processing speed, attention, and working memory. Finally, given previous studies identifying individual differences predicting variability in cognitive performance, we also explored the relationship between self-reported sleep and ratings of alertness.

## 2 Materials and methods

### 2.1 Participants

**FIGURE 1:**
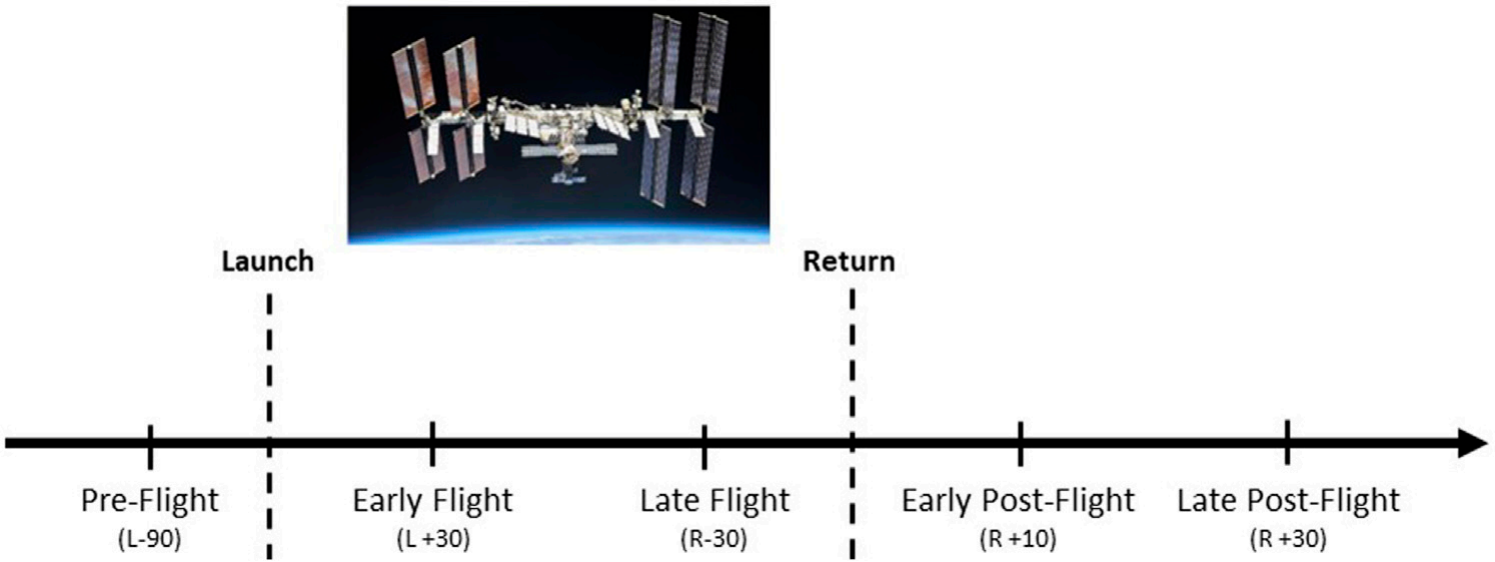
Administration Protocol of the Cognition Battery on 6-month ISS missions. Note: Pre-flight assessment occurred approximately 90 (*M* = 116.44, *SD =* 65.42) days before launch (L). The early flight assessment occurred approximately 30 (*M* = 28.88, *SD* = 10.01) days after launch and late flight occurred approximately 30 days before return (R). Early post-flight occurred approximately 10 (*M* = 9, *SD* = 1.85) days after return and late post-flight was approximately 30 (*M* = 29.8, *SD* = 5.6) days after return. Photography Credit: NASA.

A total of 25 professional astronauts aged 33 to 61 (mean (SD) = 45.12 (7.25); 32% Female) participated in the NASA Spaceflight Standard Measures study. All US orbital segment astronauts assigned to a mission to the ISS were eligible to participate in the study; no additional exclusion or inclusion criteria were applied. Ten crew members had previous flight experience that ranged from 12 to 199 days inflight (mean (SD) = 129.9 (85.6)) prior to the current mission. Cognitive performance was assessed once pre-mission, twice in-mission (within 30 days of launch and return, respectively), and twice post-mission (within 10 and 30 days of landing, respectively). All crew members completed an approximately 6-month ISS mission. See Figure 1 for data collection timeline.

### 2.2 The Cognition Battery

The Cognition Battery is a computerized test that was developed to assess a range of cognitive domains in high performing individuals over successive administrations (Basner et al., 2015; Lee et al., 2020). There are 10 subtests that are modeled after well-known neuropsychological tests. The battery has 15 alternate versions and published corrections to minimize practice effects with successive administrations (Basner et al., 2020a). The Cognition Battery was installed and administered to crew on Hewlett Packard Z-Book 15 G^2^ laptops for all sessions. The average to complete a full session was 37.10 min (*SD* = 25.16). Performance metrics were calculated consistent with previous studies using the Cognition Battery in spaceflight or spaceflight analogs to facilitate comparison across studies. Metrics across most subtests are broadly characterized with speed (i.e., reaction time recorded to nearest millisecond) and accuracy scores. A familiarization and practice session preceded all pre-flight data collection session to introduce participants to the battery.

The Visual Object Learning Task (VOLT) is a task of visual learning and memory. Participants are directed to memorize three-dimensional figures and are later instructed to select those targets from a set that includes both old and new figures.

The Fractal 2-Back (F2B) is a task of nonverbal working memory. The task presents sequential figures that have the potential to repeat multiple times. Participants must respond when the current stimulus matches the one displayed two figures prior.

Abstract Matching (AM) is a difficult executive functioning task that assesses abstraction, problem-solving, and concept formation. Participants are presented with object pairs and are required to match target items to one of the two pairs based on abstract rules not explicitly provided to them.

The Line Orientation Task (LOT) is a measure of visuospatial orientation. Participants are presented with two lines and are required to rotate one until it is parallel to its counterpart. The presented lines vary in length and orientation.

The Emotion Recognition Task (ERT) assess facial emotion recognition. Participants are shown photographs of people portraying a range of facial emotions and are required to select one of five labels that most accurately describes the expressed emotion. The possible labels are “happy”, “sad”, “angry”, “fearful”, and “no emotion”.

The Matrix Reasoning Task (MRT) is a task of nonverbal abstract reasoning and pattern recognition and is analogous to a well-known paradigm often utilized for assessing general intelligence. A pattern series is presented on a grid and one item in the pattern is missing. The participant is directed to select the item that fits the pattern from a set of potential options.

The Digit Symbol Substitution Task (DSST) measures complex scanning, visual tracking, and speed of processing. Participants are provided with a legend linking single digits to unique symbols. One of the nine symbols appear on the screen and participants select the corresponding number as quickly as possible. The entire task has a fixed duration of 90 s.

The Balloon Analog Risk Test (BART) is a measure of risk-taking behavior. Participants either inflate an animated balloon or choose to collect a reward. However, the balloon will pop after an unknown number of pumps that changes from trial to trial, which requires the participant to judge the behavior of balloons and adjust their strategy. Rewards are given as a function of when it was collected relative to the balloon’s final possible size.

The Psychomotor Vigilance Test (PVT) is a task of vigilant and sustained attention that assesses response times to visual stimuli that are seen at random intervals. Crew members are asked to press the space bar as quickly as possible when they see a millisecond counter appear on the screen. They are instructed to inhibit responses when the number counter is not present. The current implementation utilized a validated 3-min version with 2–5 s interstimulus intervals and a 355-m lapse threshold.

The Motor Praxis Task (MPT) assesses sensorimotor speed. Participants click randomly generated squares on the screen that become successively smaller over the trial. Speed is assessed by time to click the squares.

### 2.3 Cognitive performance

Speed and accuracy scores were calculated for each subtest in the Cognition Battery (Basner et al., 2015; Basner et al., 2021a; Basner et al., 2020a; Basner et al., 2021b; Lee et al., 2020). Speed outcomes for each task are represented as the average response time (in milliseconds), except for the PVT, which was calculated as 10 minus the reciprocal of the response time. Accuracy is measured as the proportion correct to total stimuli for five of the subtests (VOLT, AM, ERT, MRT, DSST). For F2B, the accuracy score was a proportion that accounts for the number of matches (i.e., correct hits) and non-matches (i.e., correct rejection). The LOT accuracy score is a proportion that accounts for the number of clicks the participant was off from the correct orientation. The PVT accuracy score is a proportion that accounts for lapses, false starts, and the total number of stimuli presented. The BART risk score is a proportion that considers the maximum pumps possible and the total pumps taken. Scores range from 0 to 1 with higher values indicating greater risk tolerance. In other words, higher scores suggest that the individual is more willing to risk popping the balloon in return for a higher reward. No accuracy score was calculated for the MPT as directions for this task does not emphasize accurate performance. Published corrections to account for stimulus set and practice effects due to repeated administrations over an average of 12 days were applied (Basner et al., 2020a). Descriptive information and figures of raw scores at each mission phase can be found in Supplementary Material.

All individual outcome scores were z-transformed using the mean and standard deviation of the full sample’s baseline test session, after excluding the individual, that occurred during the pre-fight mission phase. Z-scores for speed were multiplied by (−1), such that higher z-scores indicate better (i.e., faster) performance. Higher z-scores for the BART Risk score indicates greater risk tolerance. Finally, summary speed and accuracy z-scores were calculated by averaging all subtest speed and accuracy z scores (Basner et al., 2021a; Basner et al., 2021b). While the BART speed score was included in the overall speed score, the BART risk score was not included in the overall accuracy score as it is not a measure of accuracy.

### 2.4 Surveys

Immediately prior to the first subtest, crew members were asked to self-report the number of hours of sleep they obtained the previous evening. They also provided subjective ratings of alertness from 0 (Tired) to 10 (Alert) in the moment in which they submitted their ratings (Basner et al., 2015).

### 2.5 Analytical approach

Descriptive statistics for speed and accuracy metrics for each subtest were generated for baseline (i.e., pre-flight) raw scores and for z scores at each mission phase (aim 1). A series of linear mixed model (LMM) analyses with random subject intercepts were used to assess the relationship between mission phases and each speed and accuracy performance outcome variable (aim 2). Age, gender, and previous flight experience were included as fixed effects to statistically control for reported associations with cognitive performance (Lee et al., 2020) and possible differences in the magnitude of structural brain alterations (Hupfeld et al., 2022) between novice and repeat flyers. For each LMM, residuals were plotted and normality was assessed using the Shapiro-Wilk test. Robust LMMs were utilized when violations of normality were detected (Koller, 2016). The False Discovery Rate method was applied to adjust for 21 multiple comparisons. Trimmed models with adjusted *p*-values for each measure are reported in text and tables; unadjusted *p-*values can be found in Supplementary Table S1. Follow up LMMs with random subject intercepts to facilitate pairwise comparisons included timepoint as a categorical variable to examine differences between mission phases. Tukey’s HSD was applied to adjust for Type I error and adjusted *p*-values are reported. To examine the frequency of low scores during flight and post-flight performance (aim 3), the percent frequency of data points representing performance 1.5 standard deviations at or below the baseline (i.e., pre-flight) sample mean (z ≤ −1.5) was calculated for each subtest. This cutoff was utilized because it is commonly used to detect impairment in clinical populations (Petersen et al., 2001; Petersen and Morris, 2005; Petersen et al., 1999; Jak et al., 2009). For exploratory analyses, LMMs with random subject intercepts were utilized to assess the relationships between survey items and all speed and accuracy measures for each subtest across all mission phases. Statistical significance was set to α = 0.05 and all analyses were performed using R 4.2.1.

## 3 Results

Data were downloaded, processed, visualized, and inspected for quality. Data from four subtests were excluded from data analysis, not necessarily from the same individual: one was excluded due to technical difficulties that interfered with task performance, two were excluded due to suspected non-adherence (i.e., pattern of accuracy and speed outliers indicative of rapid responding rapid and/or careless responding and elevated false positives; (Basner et al., 2015)), and one was excluded due to a participant comment indicating confusion with subtest instructions. Table 1 displays raw baseline (i.e., pre-flight) scores used to generate z-scores for the present analysis and can be used as a preliminary normative database for future studies. Descriptive information for each subtest’s z-scores by mission phase is presented in Table 2.

**TABLE 1:** Descriptive Characteristics of pre-flight baseline raw subtest scores at pre-flight mission phase.

Subtest	N	Mean	Median	SD	Min	Max
VOLT Speed	25	1627.96	1506	617.83	712.20	3097.40
VOLT Accuracy	25	.95	.95	.06	.78	1.0
F2B Speed	24	552.94	530.80	60.95	469.40	670.0
F2B Accuracy	24	.92	.93	.07	.77	1.0
AM Speed	25	2353.32	2179.6	877.69	1028.0	4587.1
AM Accuracy	25	.81	.84	.11	.60	1.0
LOT Speed	24	4691.30	4455.30	1183.95	2874.20	7026.1
LOT Accuracy	24	.78	.81	.08	.58	.97
ERT Speed	25	2645.30	2631.82	886.40	1439.60	4720.38
ERT Accuracy	25	.72	.72	.11	.47	.92
MRT Speed	25	8546.15	8021.43	2340.55	5114.22	13,908.73
MRT Accuracy	25	.79	.83	.14	.49	0.99
DSST Speed	25	1209.38	1184.2	145.95	932.9	1485.1
DSST Accuracy	25	.98	.98	.02	.95	1
BART Speed	25	637.83	575.44	429.65	0	1702.88
BART Risk	25	.72	.74	.11	.43	.93
PVT Speed	24	5.25	5.21	.24	4.75	5.73
PVT Accuracy	24	.97	.97	.04	.81	1
MPT Speed	25	1061.18	1040.5	153.53	855.5	1434.3

Note: BART Accuracy score reflects risk taking propensity. N represents number of unique data points in pre-flight baseline sample. Data points removed for F2B, PVT, and LOT, as described in Results section; SD, standard deviation.

**TABLE 2:** Descriptive Characteristics of Z-scores by subtest and mission phase.

Subtest	Pre-flight M (SD) range	Early flight M (SD) range	Late flight M (SD) range	Early post-flight M (SD) range	Late post-flight M (SD) range
Summary Speed	−.02 (.59) −1.53–1.37	−.33 (.54) −1.38–1.25	−.18 (.61) −1.07–1.38	−.13 (.64) −1.84–.95	−.04 (.5) -.86–1.15
Summary Accuracy	−.03 (.43) −.98–.76	−.07 (.41) −1.21–.34	−.06 (.37) −1.09–.57	−.20 (.44) −1.56–.56	−.22 (.44) −1.34–.48
VOLT Speed	−.02 (1.09) −2.79–1.59	−.10 (1.17) −3.74–1.60	.09 (.79) −1.71–1.43	.07 (.91) −3.12–1.42	.004 (.77) −2.14–1.27
VOLT Accuracy	−.04 (1.14) −3.19–.80	−.13 (1.04) −3.07–.80	−.21 (.85) −2.03–.80	.03 (.86) −1.55–.80	−.52 (1.10) −2.80–.80
F2B Speed	−.01 (1.07) −2.15–1.46	−1.24 (1.64) −4.87–1.46	−1.06 (1.59) −4.23–1.40	−.82 (1.4) −3.94–1.40	−1.24 (1.41) −5.25–.57
F2B Accuracy	−.02 (1.08) −2.55–1.18	.24 (.76) −1.03–1.18	.01 (.97) −2.23–1.18	−.15 (.95) −1.94–1.18	−.16 (.96) −2.29–1.18
AM Speed	−.02 (1.10) −3.06–1.62	.10 (.87) −1.83–1.31	.38 (.79) −1.12–1.59	.45 (.71) −1.19–1.76	.50 (.52) -.95–1.37
AM Accuracy	−.01 (1.07) −2.17–1.87	−.42 (1.38) −3.56–1.69	−.09 (1.01) −2.07–1.87	−.38 (1.4) −4.03–1.41	−.55 (1.27) −3.03–1.39
LOT Speed	−.01 (1.07) −2.22–1.66	−.15 (1.06) −3.11–1.96	−.24 (.97) −2.15–1.48	−.11 (.95) −1.95–1.50	−.14 (.87) −2.11–1.24
LOT Accuracy	−.01 (1.13) −2.78–2.74	.29 (.9) −1.64–1.34	.14 (.87) −2–2.33	−.13 (1.0) −2.36–1.61	.06 (1.09) −2.69–1.67
ERT Speed	−.02 (1.10) −2.73–1.45	.03 (.88) −2.12–1.06	.12 (.92) −1.73–1.44	.40 (.74) −1.15–1.72	.39 (.69) −1.69–1.45
ERT Accuracy	−.01 (1.08) −2.50–2.08	−.23 (.70) −1.41–.85	−.04 (1.15) −2.10–1.96	−.10 (1.13) −2.83–2.07	.31 (.99) −1.79–1.81
MRT Speed	−.01 (1.08) −2.66–1.57	−.33 (1.09) −2.65–1.49	−.19 (1.18) −2.79–2.03	.13 (1.20) −2.16–2.02	.41 (1.07) −2.24–2.29
MRT Accuracy	−.02 (1.09) −2.47–1.63	−.29 (.85) −2.07–1.52	−.45 (1.03) −3.31–1.52	−.40 (1.17) −3.03–1.55	−.38 (1.01) −2.48–.93
DSST Speed	−.01 (1.08) −2.10–2.10	−.79 (1.54) −5.58–1.71	−.73 (1.48) −3.72–2.72	−.75 (1.72) −6.44–1.62	−.33 (1.39) −4.30–2.34
DSST Accuracy	−.01 (1.07) −2.19–.97	.04 (1.17) −.341–.97	.22 (1.11) −2.80–.97	−.20 (1.38) −3.77–.97	−.30 (1.58) −4.96–.97
BART Speed	−.01 (1.09) −2.95–1.59	−.11 (.95) −2.44–1.59	.01 (.90) −1.72–1.22	−.19 (.75) −2.09–1.24	.02 (.80) −1.94–1.59
BART Risk	−.02 (1.12) −3.35–2.13	−.20 (1.19) −3.57–1.80	−.88 (.95) −3.08–.47	−.72 (1.02) −3.81–.99	−.96 (1.05) −3.27–1.09
PVT Speed	−.003 (1.08) −2.22–2.28	−.53 (1.04) −2.76–1.08	−.23 (1.43) −4.12–2.01	−.39 (1.45) −4.37–2.03	−.40 (1.73) −4.64–2.71
PVT Accuracy	−.11 (1.48) −6.36–.76	−.09 (.68) −1.52–.75	−.06 (.61) −1.38–.75	−.29 (1.06) −3.56–.76	−.19 (1.03) −4.12–.73
MPT Speed	−0.02 (1.09) −2.87–1.42	−.16 (.99) −2.95–1.46	.02 (.87) −2.06–1.53	.12 (1.18) −3.79–1.34	.41 (.89) −1.76–1.94

Note: Z-scores calculated with current samples pre-flight baseline raw scores. BART Accuracy score reflects risk taking propensity; SD, standard deviation.

### 3.1 Cognitive performance over mission phases

**FIGURE 2:**
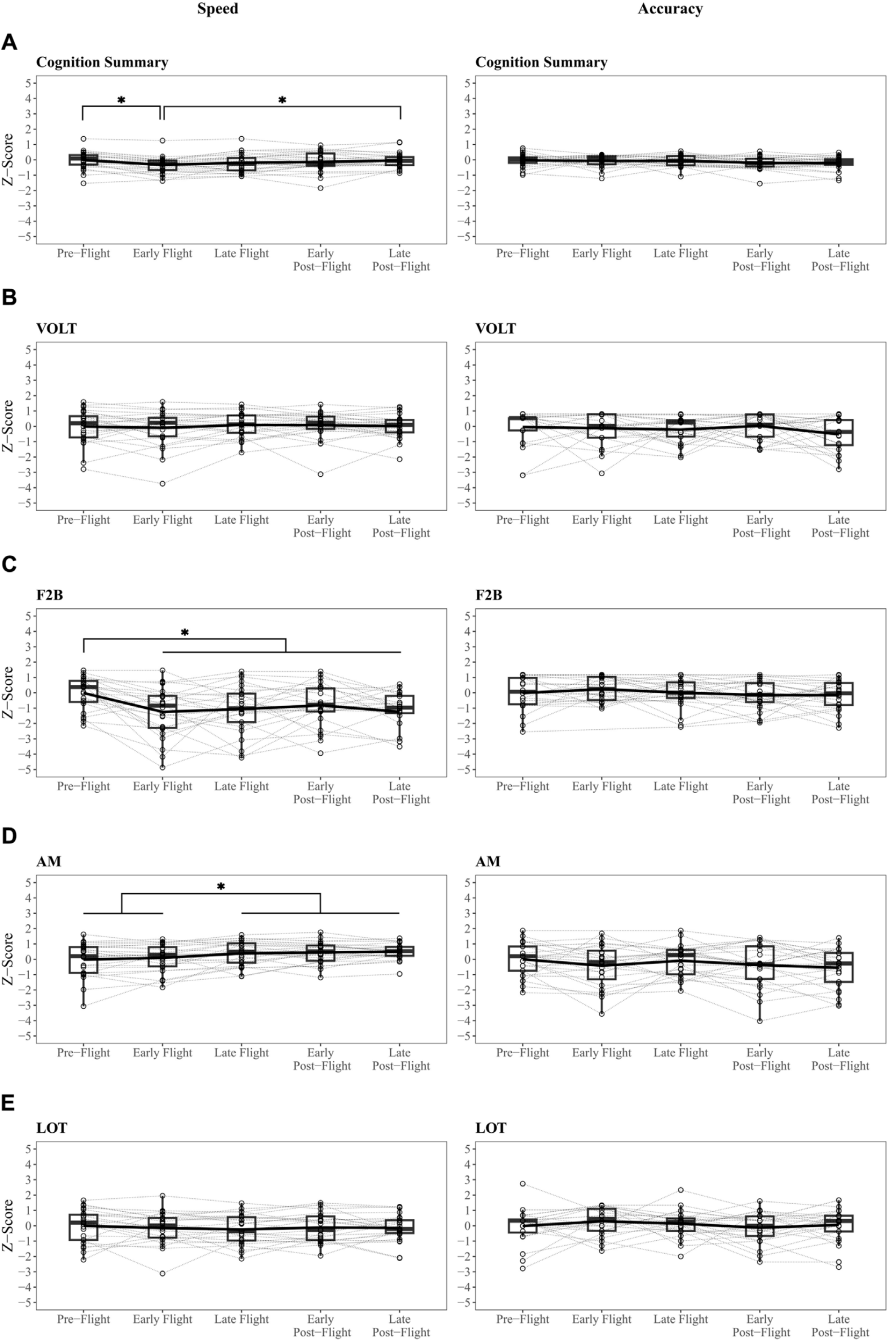
Speed and Accuracy Scores Over Mission Phase. Adjusted pairwise comparisons of speed and accuracy scores between mission phases in a subset of Cognition Battery subtests. **(A)** Summary speed and accuracy scores remained stable. **(C)** Speed was slower on the Fractal 2 Back (F2B) task at the early flight mission phase and persisted through post-flight phases. **(D)** Performance on Abstract Matching (AM) was faster over time, likely reflecting residual practice effects. **(B)** and **(E)** Stable scores across mission phases were observed on the Visual Object Learning (VOLT) and Line Orientation Task (LOT). *adjusted *p* < .05.

Hypothesis 1 suggested that there would be mild decrements in early flight and early post-flight mission phases relative to pre-flight. For each outcome we first present results assessing linear relationships of performance over mission phases (see Supplementary Table S1) followed by direct pairwise comparisons between mission phases. Linear mixed models revealed no linear relationship between mission phase and the summary speed score. In support of Hypothesis 1, pairwise analysis characterizing differences in summary speed between mission phases (Figure 2, Panel A) revealed significant, but small, differences between pre-mission and early flight (*β* = .35, adjusted *p* = .002, ∆z = .31) as well as early flight and late post-flight (*β* = −.33, adjusted *p* = .004, ∆z = .29). There was no significant difference between pre-flight and early post-flight. Summary accuracy declined over time (*β* = −.05, adjusted *p* = .03) but no pairwise comparisons were significantly different (Figure 2, Panel A) and the effect sizes of the mean differences between mission phases were negligible.

**FIGURE 3:**
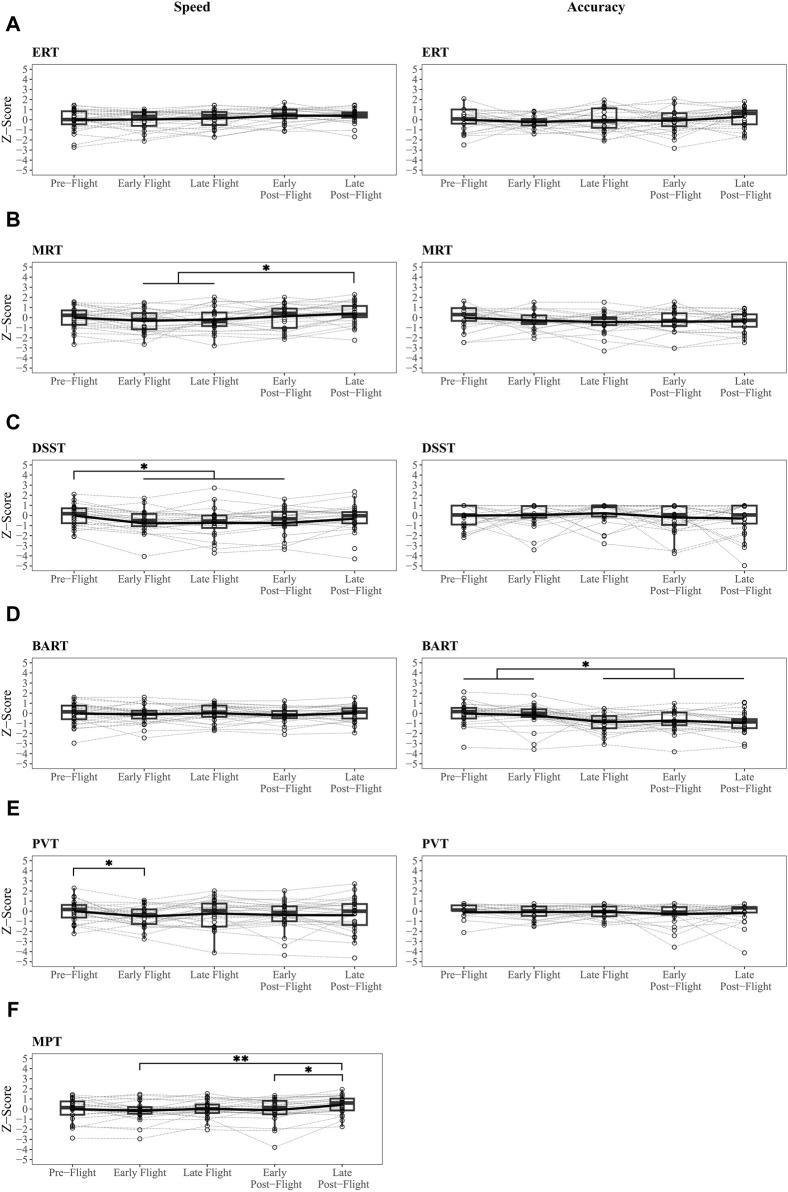
Speed and Accuracy Scores Over Mission Phase Continued. Adjusted pairwise comparisons of speed and accuracy scores between mission phases in a subset of Cognition Battery subtests. **(A)** Stable scores across mission phases on the Emotion Recognition Task (ERT). **(B)** Performance on Matrix Reasoning Task (MRT) was faster over time, likely reflecting residual practice effects. **(C)** Speed was slower on the Digit Symbol Substitution Task (DSST) at the early flight mission phase and persisted through the early post-flight phase. **(D)** Reduced risk taking propensity on the Balloon Analog Risk Test (BART) task after the early flight phase. **(E)** Reduced speed the Psychomotor Vigilance Test (PVT) during the early flight phase only. **(F)** Faster performance on Motor Praxis Task (MPT) during the late post mission phase. *adjusted *p* < .05.

Support for Hypothesis 1 across the 10 subtests in the Cognition battery varied by domain. Differences between mission phases were observed in several subtests, even among those with no linear associations with time (Figures 2, 3). VOLT speed and accuracy remained stable over time and between mission phases. No pairwise comparisons between mission phases survived multiple comparisons and the effect sizes of the mean differences in VOLT accuracy (∆z’s = .07 to .55) and speed (∆z’s = .02 to .11) between mission phases ranged from negligible to medium. See Figure 2, Panel B.

F2B speed declined over time (*β* = −.23, 95% CI [-.35–.1], adjusted *p* < .001). Pairwise contrasts revealed faster performance during pre-mission compared to all other mission phases (*β*′s range .8–1.29; adjusted *p*’s range .01 to < .001). The effect sizes of the significant mean differences between mission phases were large (∆z’s = .81 to 1.23). F2B accuracy remained stable over time and between mission phases. See Figure 2, Panel C.

AM speed improved over time (*β* = .12, 95% CI [.07 – .17], adjusted *p* < .001). Pre-flight performance was slower than late in-flight (*β* = −.35, adjusted *p* = .03, ∆z = .40) and both post-flight phases (vs early: *β* = −.39, adjusted *p* = .01, ∆z = .47; vs. late: *β* = −.43, adjusted *p* = .004; ∆z = .52). Early in-flight performance was also slower than both post-flight phases (vs early: *β* = −.36, adjusted *p* = .03; ∆z = .35; vs late: *β* = −.41, *p* = .009; ∆z = .40). The effect sizes of the significant mean differences ranged from small to medium. AM accuracy remained stable over time and between mission phases. See Figure 2, Panel D.

LOT speed and accuracy remained stable over time and between mission phases. See Figure 2, Panel E.

ERT speed improved over time (*β* = .1, 95% CI [.03 – .17], adjusted *p =* .02). No pairwise comparisons between mission phases were significant and the effect sizes of the mean differences for ERT speed between mission phases ranged from negligible to small (∆z’s = .05 to .41). ERT accuracy remained stable over time and between mission phases. See Figure 3, Panel A.

MRT speed improved over time (*β* = .13, 95% CI [.04 – .23], adjusted *p =* .02). Late post-flight performance was faster relative to both in flight mission phases (vs early: *β* = −.81, adjusted *p* = .002, ∆z = .74; vs late: *β* = −.6, *p* = .04, ∆z = .60); effect sizes of the significant mean differences between mission phases ranged from medium to large. MRT accuracy remained stable over time and between mission phases. See Figure 3, Panel B.

There was no significant linear association between time and DSST speed (*β* = −.05, 95% CI [-.13–.04], adjusted *p* = .42). However, comparison of discrete mission phases revealed slowed performance during both in flight (vs early: *β* = .78, adjusted *p* = .0001, ∆z = .78; vs late: *β* = .73, adjusted *p* = .0002, ∆z = .72) and early post-flight (*β* = −.61, adjusted *p* = .003, ∆z = .74) compared to pre-flight performance. Effect sizes of the significant mean differences between mission phases were large. DSST accuracy remained stable over time and between mission phases. See Figure 3, Panel C.

BART speed remained stable over time and between mission phases. The BART Risk Score declined over time (*β* = −.23, 95% CI [-.31 to −.15], adjusted *p* < .001). Pairwise comparisons between mission phases revealed greater risk tolerance during pre-flight and early flight phases compared to late flight (vs pre-flight: *β* = .78, adjusted *p* = .0002, ∆z = .86; vs early flight: *β* = .70, adjusted *p* = .001, ∆z = .68), early post-flight (vs pre-flight: *β* = .61, adjusted *p* = .007, ∆z = .70; vs early flight: *β* = .53, adjusted *p* = .03, ∆z = .52) and late post-flight (vs pre-flight: *β* = .9, adjusted *p* < .0001, ∆z = .94; vs early flight: *β* = .82, adjusted *p* = .0001, ∆z = .77) mission phases. The effect sizes of the significant mean differences between mission phase ranged from medium to large. See Figure 3, Panel D.

There was no significant relationship between time and PVT speed (*β* = −.07, 95% CI [-.17–.03], adjusted *p* = .27). However, comparison of discrete mission phases revealed slowed performance during early flight compared to pre-flight mission phases (*β* = .62, adjusted *p* = .05, ∆z = .5); the effect size of the mean difference was medium. PVT accuracy remained stable over time and between mission phases. See Figure 3, Panel E.

MPT speed improved over time (*β* = .09, 95% CI [.02 – .17], adjusted *p* = .03). Pairwise comparisons of mission phases revealed that MRT speed was faster in the late post-flight mission phase compared to early in-flight (*β* = −.63, adjusted *p* = .0005, ∆z = .57) and early post-flight (*β* = −.48, adjusted *p* = .02, ∆z = .29) and marginally faster than late flight (*β* = −.4, adjusted *p* = .06, ∆z = .39). Corresponding effect sizes for the significant mean differences between mission phases were small to moderate. See Figure 3, Panel F.

### 3.2 Frequency of low scores

**FIGURE 4:**
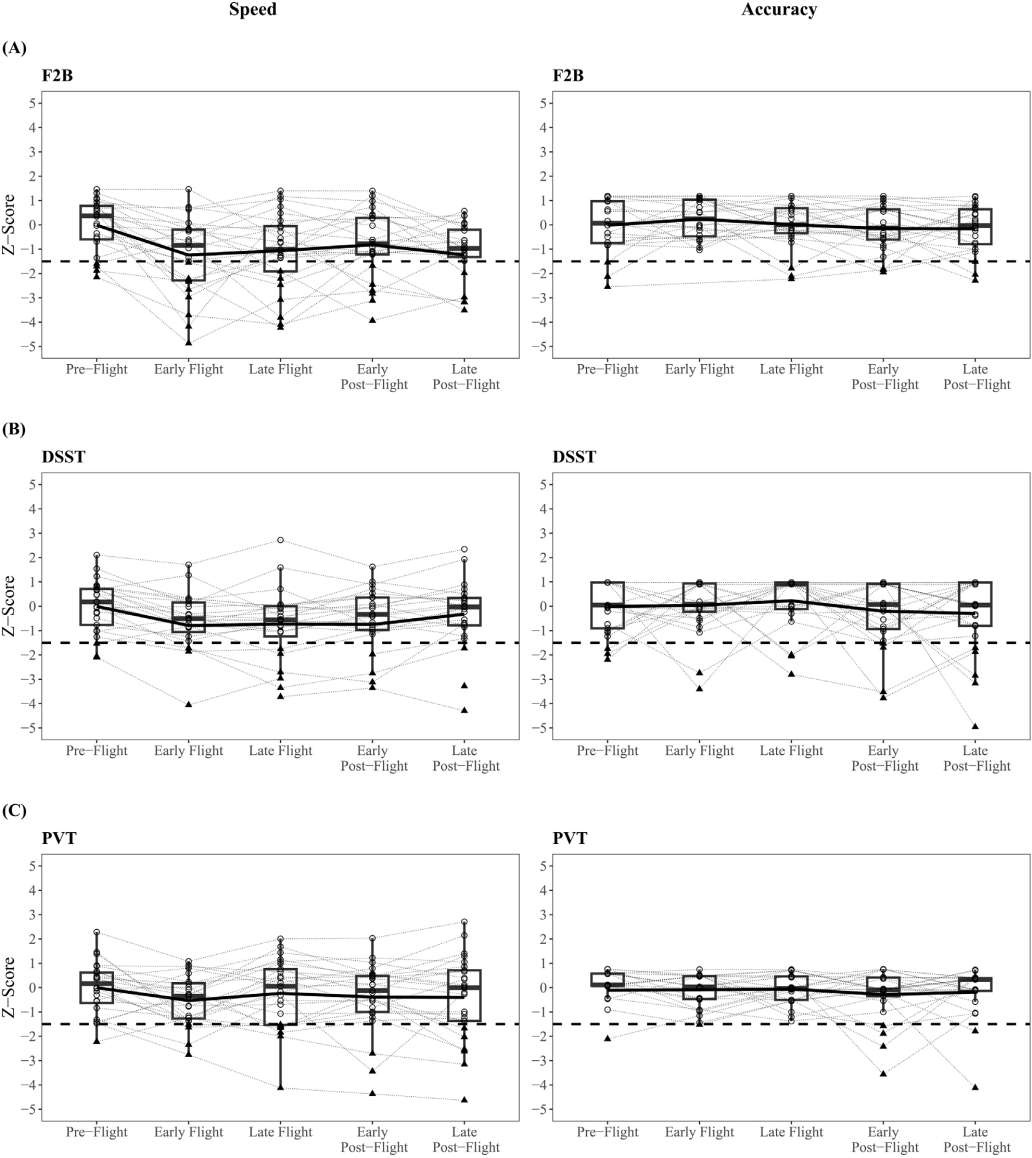
Frequency of Low Scores Low scores were defined as any individual test score that was at or below 1.5 standard deviations of the samples pre-flight baseline performance (z-score ≤ −1.5). The Fractal 2-Back (F2B; **(A)**), Digit Symbol Substitution Task (DSST; **(B)**), and Psychomotor Vigilance Test (PVT; **(C)**) subtests had greater than 13% of flight and post-flight data that were classified as a low score. All low scores that were below our pre-determined cut-off (dotted line) are represented in solid triangles.

Overall, 11.8% of all individual test scores were at or below 1.5 standard deviations of the full sample baseline mean (i.e., z-score ≤ −1.5). Consistent with Hypothesis 2, the subtests with the greatest frequency of low scores were on tasks of working memory (F2B; 18.7%), processing speed (DSST; 16.7%), and sustained attention (PVT; 13.6%). The frequency of low scores on the BART risk score (19.2%) was also higher than speed and accuracy scores on other subtests; these scores represent individual observations indicating substantially decreased risk taking propensity compared to pre-flight baseline. See Table 3. Figure 4 selects the three subtests with the highest frequencies of low scores, respectively, and presents data across mission phases.

**TABLE 3:** Percent frequency of low scores during flight and post-flight.

Subtest	Speed	Accuracy	Subtest total
VOLT	5.1	13.1	9.1
F2B	28.3	9.1	18.7
AM	1.01	20.2	10.6
LOT	7.1	7.1	7.1
ERT	5.1	8.1	6.6
MRT	11.1	13.1	12.1
DSST	19.4	14.3	16.7
BART	7.1	19.2	13.3
PVT	20.2	7.1	13.6
MPT	8.1	--	8.1
Total	11.2	12.4	11.8

Note: Low scores were identified as scores at or below 1.5 standard deviations below average baseline (i.e., pre-flight) performance (z ≤ -1.5). BART accuracy score reflects risk taking propensity.

### 3.3 Sleep duration and alertness over mission phases

Across all mission phases, there were no relationships between hours of sleep reported and performance on any subtest of the Cognition battery. Alertness was inversely related to DSST accuracy (*β* = −.1, *p* = .05) only. See Table 4.

**TABLE 4:** Self-reported hours of sleep and alertness.

	Pre-flight M (SD) range	Early flight M (SD) range	Late flight M (SD) range	Early post-flight M (SD) range	Late post-flight M (SD) range
Sleep	6.53 (1.56) .75–8.5	6.42 (.94) 4–8	6.38 (1.21) 3–8	7.24 (1.23) 3.5–9	6.98 (.7) 6–8
Alert	6.56 (2.02) 2–9	6.46 (1.89) 3–10	5.96 (2.46) 2–9	5.92 (2.25) 2–9	6.32 (2.1) 3–10

Note: Self-reported number of hours slept during the night prior to cognitive performance testing. Subjective ratings of alertness assessed on a 10-point scale, from (0) Tired to 10 (Alert), per (Basner et al., 2014). SD, standard deviation.

## 4 Discussion

This investigation characterized performance across a wide breadth of cognitive domains in a sample of 25 astronauts on 6-month ISS missions. We were unable to locate any published studies with a more comprehensive or larger dataset on a sample of astronauts; thus, we believe this study makes a substantial contribution in characterizing astronaut cognitive performance in spaceflight. We have reported pre-flight baseline descriptive information characterizing cognitive performance across all subtests that can be utilized as a preliminary normative database for future research. As a group, astronaut crew demonstrated generally stable performance on summary measures of cognition and only mild changes in isolated cognitive domains during specific mission phases. Specifically, slowed performance was observed early in flight on tasks of processing speed (i.e., DSST), visual working memory (i.e., F2B), and sustained attention (i.e., PVT); slowed processing speed and working memory persisted into post-flight. Risk taking propensity also reduced after the early flight mission phases. A total of 11.8% of all flight and post-flight scores were classified as low, such that they fell at or below 1.5 standard deviations below the sample’s pre-flight baseline mean. Finally, exploratory analysis found no consistent relationships between cognitive performance and either self-reported amount of sleep or ratings of alertness. This study makes three important contributions which are discussed next.

First, we report no systematic decline in cognitive performance during 6-month low earth orbit missions among the largest sample of professional astronauts published to date. This is in contrast to some of the previous research that observed declines across several cognitive domains during flight and post-flight mission phases (Garrett-Bakelman et al., 2019; Takács et al., 2021). Our results do not necessarily contradict these findings as we also observed interindividual variability in our sample and it is possible that longer duration missions elicit greater decrements at later mission phases. However, we did not find support for group level impacts of spaceflight conditions on cognitive performance in low earth orbit.

Next, by using a comprehensive assessment of cognitive functions we identified cognitive domains that were more variable across phases of a 6-month low earth orbit missions. This suggests that these cognitive domains are more vulnerable to spaceflight conditions than other domains. Specifically, we observed early flight slowed performance, but stable accuracy, on tasks of processing speed and visual working memory that persisted through the duration of the mission. Visual working memory speed did not return to pre-flight levels at 30 days post mission. Slower performance on the PVT was observed during early flight only. Notably, these decrements were present despite generally preserved, or even improved, sensorimotor functioning. These findings are consistent with astronaut self-report of “brain fog” (Clément et al., 2020; Stuster, 2010; Stuster, 2016), the NASA Twin Study (Garrett-Bakelman et al., 2019), and analog studies simulating prolonged microgravity (Basner et al., 2021a; Basner et al., 2021b; Liu et al., 2012; Lipnicki and Gunga, 2009) and sleep restriction (Wang et al., 2014). This is also consistent with documented pre to post mission alterations in brain structure and function (Roy-O’Reilly et al., 2021; Doroshin et al., 2022; Jillings et al., 2023) that can contribute to cognitive inefficiency. Our results also revealed decreases in the BART risk taking score after the early flight mission phase, suggesting that astronauts were less likely to take risks in late mission and post mission phases. Greater comfort with risk taking at early flight may reflect the novel and inherently hazardous nature of operational activities that require crew to accept risk in order to complete. Risk taking is relatively understudied in spaceflight despite the hazardous environment, though others have reported increases in flight (Garrett-Bakelman et al., 2019), no changes during simulated prolonged microgravity (Basner et al., 2021a; Basner et al., 2021b), and decreases during social isolation (Erskine-Shaw et al., 2017; Zivi et al., 2023) and fatigue (Hisler and Krizan, 2019). This finding warrants further study as the ability to modulate risk taking propensity across the mission may be an adaptive skill that enables the astronaut to appropriately approach mission demands. Finally, we observed no decrements in concept formation, abstract reasoning, or visuospatial learning and memory, though other studies indicate there may be individual differences in vulnerability to decrements in these domains (Premkumar et al., 2013; Garrett-Bakelman et al., 2019). Indeed, frontal and hippocampal structures that support these functions are sensitive to radiation exposure (Desai et al., 2022), stress (Mandrick et al., 2016; Wingenfeld and Wolf, 2014), isolation and confinement (Stahn et al., 2019), and microgravity (Koppelmans et al., 2016; Lee et al., 2021; Li et al., 2015). Thus, it is possible that longer duration missions with greater exposure to spaceflight hazards or individual vulnerabilities to stressors will exacerbate decrements in future exploration missions.

Finally, we present the first published professional astronaut preliminary normative dataset for the Cognition Battery that can be used to derive standard scores to identify significant deviations from pre-flight baseline functioning. For example, despite modest group level differences in cognitive performance across most subtests, 11.8% of observations across all tasks during flight or post-flight fell at or below 1.5 standard deviations of the samples pre-flight baseline mean. The greatest number of observations below cut off were on tasks assessing working memory, processing speed, and sustained attention, which are domains vulnerable to state-like alterations related to acute changes in sleep (Jones et al., 2022; Lim and Dinges, 2010), stress (Eysenck and Calvo, 1992), workload (Ghalenoei et al., 2022), and dehydration (Wittbrodt and Millard-Stafford, 2018). Though there is no evidence of an increased frequency of low scores in this sample compared to other cognitively healthy populations (Crawford et al., 2007; Binder et al., 2009), even highly infrequent or temporary barriers to adequately engaging cognitive functions required to perform complex operational task may result in catastrophic consequences in spaceflight. This preliminary normative dataset provides operational support with the ability to identify low scores during flight, elucidate effective in mission support to enhance performance (i.e., stress reduction, timeline alterations), or inform concept-of-operations during mission critical tasks. This dataset can also be utilized in future research to compare performance against future astronauts on extended duration missions or those that venture beyond low earth orbit and include more extreme stressors.

These significant contributions underscore critical knowledge gaps that should be prioritized for future research. First, there are likely individual differences in susceptibility to spaceflight environmental stressors. The current sample size was too small to stratify our normative baseline by age. Although we did not find clear associations between cognitive performance and self-reported hours of sleep or ratings of alertness, there are several other stressors that are not accounted for in the current dataset such as workload, stress and fatigue specific to extravehicular activities, nutrition/exercise, and individual behavioral health. These may become more impactful during future space exploration missions as conditions of greater radiation exposure, extra-vehicular surface activities, and earth independent operations deplete individual cognitive reserve to unmask larger decrements. The operational consequences to observed changes in cognitive performance also remains unknown. The everyday demands required of spaceflight are more complex, dangerous, and uncertain than those of terrestrial life suggesting that current clinical thresholds to define impairment may not be appropriate. Basner et al. (2020b) reported associations between a spaceflight docking task and processing speed, sustained attention, visuospatial orientation, and abstract reasoning, suggesting that this mission critical task is vulnerable to some of the decrements we observed in this investigation. Future studies investigating the relationship between cognitive performance and other operationally activities are required to determine appropriate decrement thresholds beyond which performance is most at risk. Further development of earth independent operational supports to maintain optimal cognitive performance will further reduce the risk to individual cognitive health and performance on operational tasks.

The reported results should be interpreted in the context of some limitations. Despite applying corrections for practice effects, the reported data suggests continued improvement over time in a subset of the Cognition Battery subtests. This likely due to small sizes and key differences between our study design and that of the published corrections, including administration intervals (i.e., 10–28 days vs > 60 days) and population (i.e., university vs. operational settings). Updates to the practice corrections may be warranted now that the Cognition Battery has become more commonly used among several high performing operational environments. Cognitive performance assessed during the early flight and early post-flight stages were collected on average 28.88 (*SD* = 10.01*)* days after launch and 9 (*SD* = 1.85) days after landing. Thus, performance was not characterized during what may be the most acute transitional phases of a mission or during long term follow up. Nevertheless, the pre-flight data published here is the first preliminary astronaut normative dataset that can be used in future studies to derive standard scores for independent samples.

In summary, we observed no evidence for systematic widespread declines in cognitive performance suggestive of damage to the central nervous system during 6-month ISS missions. Our results revealed early mildly slowed performance in isolated tasks of processing speed, working memory, and attention that persisted to varying degrees through the mission, reduced risk taking after early flight mission phases, and stable performance on tasks of memory and executive functions. This investigation makes a substantial contribution by providing a pre-flight normative sample of professional astronauts that can be used to characterize individual or group level deviations from baseline functioning, characterizes cognitive performance under conditions of spaceflight in low earth orbit, and substantially shapes a growing body of literature towards a future that best positions humans to thrive in space.

## Data Availability

The data presented in the study are deposited in the NASA Life Science Portal at https://nlsp.nasa.gov/view/lsdapub/lsda_experiment/ea3997a6-1093-5e0f-8f54-47684d4f1e73.
