# Supplementary material for: Cognitive performance in ISS astronauts on 6-month low earth orbit missions
Source: Front Physiol. 2024 Nov 20;15:1451269. doi: 10.3389/fphys.2024.1451269 (PMC11614644; doi:10.3389/fphys.2024.1451269)
Supplement: Supplementary file 1 [file Table1.docx]

Supplemental Table S1. Trimmed Linear Mixed Models Assessing the Main Effect of Mission Phase on Performance.

|  | **Intercept** | | **Age** | | **Sex** | | **Flight Experience** | | **Mission Phase** | | |
| --- | --- | --- | --- | --- | --- | --- | --- | --- | --- | --- | --- |
|  | **β** | ***p*** | **β** | ***p*** | **β** | ***p*** | **β** | ***p*** | **β** | ***p*** | **Adj. *p*** |
| **Summary Speed** | .13 | .47 |  |  | **-.49** | **.02*** |  |  | .02 | .38 | .47 |
| **Summary Accuracy** | .07 | .36 |  |  |  |  |  |  | -.05 | **.01*** | **.03*** |
| **VOLT Speed** | **.61** | **.005*** |  |  | **-.75** | **.002*** |  |  | .01 | .84 | .88 |
| **VOLT Accuracy** | .24 | .24 |  |  |  |  |  |  | -.11 | **.05*** | **.12** |
| **F2B Speed** | -.02 | .96 |  |  |  |  |  |  | **-.23** | **<.001**** | **<.001**** |
| **F2B Accuracy** | **.84** | **.001*** |  |  | **-.8** | **.001*** |  |  | -.09 | .06 | .13 |
| **AM Speed** | -.05 | 0.77 |  |  |  |  |  |  | **.12** | **<.001**** | **<.001**** |
| **AM Accuracy** | .09 | .74 |  |  |  |  |  |  | -.1 | .04* | .12 |
| **LOT Speed** | -.03 | .88 |  |  |  |  |  |  | -.03 | .38 | .47 |
| **LOT Accuracy** | -.09 | .68 |  |  | **.7** | **<.001**** | **-.4** | **.01*** | -.03 | .63 | .70 |
| **ERT Speed** | .35 | .16 |  |  | **-.62** | **.02*** |  |  | **.1** | **.003*** | **.02*** |
| **ERT Accuracy** | -.24 | .26 |  |  |  |  |  |  | .08 | .16 | .27 |
| **MRT Speed** | -.42 | .08 |  |  |  |  |  |  | **.13** | **.005*** | **.02*** |
| **MRT Accuracy** | -.05 | .82 |  |  |  |  |  |  | -.08 | .09 | .17 |
| **DSST Speed** | -.29 | .26 |  |  |  |  |  |  | -.05 | .28 | .42 |
| **DSST Accuracy** | .22 | .33 |  |  |  |  |  |  | -.03 | .59 | .69 |
| **BART Speed** | .36 | .18 |  |  | **-.62** | **.04*** |  |  | 0 | .99 | .99 |
| **BART Risk** | .21 | .26 |  |  |  |  |  |  | **-.23** | **<.001**** | **<.001**** |
| **PVT Speed** | -.09 | .74 |  |  |  |  |  |  | -.07 | .17 | .27 |
| **PVT Accuracy** | .11 | .45 |  |  |  |  |  |  | -.04 | .33 | .46 |
| **MPT Speed** | **2.29** | **.002*** | **-.05** | **.001*** |  |  |  |  | **.09** | **.01*** | **.03*** |

Note: BART Accuracy score reflect risk taking propensity. Adjusted *p* values represented corrections for multiple comparisons using the False Discovery Rate Method. **p* < .05, ***p* < .001

Supplemental Table S2. Relationships between Pre-Flight Cognitive Performance and Age, Gender, and Previous Flight Experience

|  | **Age** | | **Sex** | | **Flight Experience** | |
| --- | --- | --- | --- | --- | --- | --- |
|  | ***r*** | ***p*** | ***t*** | ***p*** | **t** | ***p*** |
| **VOLT Speed** | -.28 | .17 | -1.49 | .15 | 1.86 | .08 |
| **VOLT Accuracy** | -.17 | .41 | .83 | .42 | .32 | .75 |
| **F2B Speed** | -.05 | .80 | .30 | .77 | .31 | .76 |
| **F2B Accuracy** | .16 | .45 | 1.80 | .10 | -.62 | .55 |
| **AM Speed** | -.05 | .83 | -1.24 | .23 | .99 | .33 |
| **AM Accuracy** | -.45 | .03* | -.40 | .70 | 1.55 | .13 |
| **LOT Speed** | .23 | .28 | -1.12 | .28 | .11 | .91 |
| **LOT Accuracy** | -.07 | .75 | -.25 | .81 | 3.16 | .007* |
| **ERT Speed** | .03 | .91 | -1.27 | .22 | 1.20 | .24 |
| **ERT Accuracy** | .31 | .13 | .72 | .48 | -1.56 | .13 |
| **MRT Speed** | -.30 | .15 | .33 | .75 | 1.07 | .30 |
| **MRT Accuracy** | .05 | .81 | -.17 | .86 | -1.64 | .12 |
| **DSST Speed** | .33 | .10 | -2.08 | .05^ | -.84 | .41 |
| **DSST Accuracy** | .10 | .63 | -.65 | .53 | -1.12 | .27 |
| **BART Speed** | -.20 | .33 | -2.13 | .05^ | .85 | .41 |
| **BART Risk** | .33 | .11 | -1.07 | .30 | -.66 | .52 |
| **PVT Speed** | -.19 | .38 | .73 | .48 | .74 | .47 |
| **PVT Accuracy** | -.004 | .99 | 1.64 | .12 | -.10 | .92 |
| **MPT Speed** | .29 | .16 | -3.26 | .003* | .17 | .87 |

Note: Raw corrected scores were utilized for these analyses. BART Accuracy score reflect risk taking propensity. *r =* Spearman’s coefficient; t = Welch’s t test. **p* < .05, ^ *p* = .05.

Supplemental Table S3. Descriptive Characteristics of Raw by Subtest and Mission Phase

| **Subtest** | **Pre-flight**  M (SD) Range | **Early flight**  M (SD) Range | **Late flight**  M (SD) Range | **Early Post-flight**  M (SD) Range | **Late post-flight**  M (SD) Range |
| --- | --- | --- | --- | --- | --- |
| **VOLT Speed** | 1627.96 (617.83) 712.20 – 3097.40 | 1683.65 (673.4) 690.8 – 3615.3 | 1567.78 (460.3) 790.7 – 2504.4 | 1582.56 (549.95) 811.1 – 3544.4 | 1621.22 (439.8) 886.7 – 2741.5 |
| **VOLT Accuracy** | .95 (.06) .78 – 1.0 | .94 (.07) .75 – 1.0 | .94 (.05) .82 – 1.0 | .95 (.05) .86 – 1.0 | .92 (.07) .78 – 1.0 |
| **F2B Speed** | 552.94 (60.95) 469.4 – 670.0 | 628.04 (97.03) 469.4 – 834.7 | 616.9 (95.39) 467.7 – 812.10 | 602.06 (83.21) 467.8 – 792.9 | 627.02 (81.66) 516.6 – 846.6 |
| **F2B Accuracy** | .92 (.07) .77 – 1.0 | .94 (.05) .84 – 1.0 | .92 (.07) .77 – 1.0 | .91 (.07) .78 – 1.0 | .91 (.07) .76 – 1.0 |
| **AM Speed** | 2353.32 (877.69) 1028.0 – 4587.1 | 2264.35 (724.74) 1246.3 – 3926.5 | 2014.07 (668.01) 1056.9 – 3364.1 | 1954.39 (575.82) 908.7 – 3163.6 | 1910.12 (417.89)1193.3 – 2985.4 |
| **AM Accuracy** | .81 (.11) .6 – 1.0 | .77 (.15) .42 – 1.0 | .80 (.10) .61 – 1.0 | .77 (.15) .41 – .97 | .75 (.13) .49 – .95 |
| **LOT Speed** | 4691.3 (1183.95) 2874.2 – 7026.1 | 4871.07 (1218.39) 2534.1 – 8341.9 | 4980.0 (1124.30) 3081.5 – 7236.5 | 4824.74 (1090.94) 2988.0 – 6937.9 | 4862.98 (997.43) 3310.7 – 7158.3 |
| **LOT Accuracy** | .78 (.08) .58 – .97 | .80 (.07) .64 – .89 | .79 (.07) .61 – .97 | .77 (.08) .58 – .89 | .78 (.09) .56 – .92 |
| **ERT Speed** | 2645.3 (886.4) 1439.6 – 4720.38 | 2621.02 (757.04) 1771.7 – 4441.09 | 2527.08 (780.23) 1430.77 – 4200.07 | 2282.83 (643.33) 1086.8 – 3667.42 | 2289.49 (573.68) 1388.8 – 3926.89 |
| **ERT Accuracy** | .71 (.11) .47 – .92 | .69 (.08) .55 – .8 | .71 (.12) .49 – .93 | .70 (.12) .44 – .94 | .75 (.11) .51 – .91 |
| **MRT Speed** | 8546.15 (2340.55) 5114.22 – 13908.73 | 9315.28 (2485.21) 5020.26 – 14327.72 | 8986.65 (2703.81) 4034.99 – 15213.68 | 8232.45 (2736.61) 3748.7 – 13313.09 | 757.36 (2405.76) 3124.24 – 13031.11 |
| **MRT Accuracy** | .79 (.14) .49 – .99 | .75 (.11) .54 – 1.0 | .73 (.14) .33 – 1.0 | .74 (.15) .42 – 1.0 | .74 (.13) .49 – .91 |
| **DSST Speed** | 1209.38 (145.95) 932.9 – 1485.10 | 1322.4 (208.59) 987.2 – 1965.4 | 1315.22 (204.36) 848.3 – 1709.6 | 1316.29 (236.28) 999.0 – 2079.9 | 1255.77 (190.77) 899.9 – 1788.6 |
| **DSST Accuracy** | .98 (.02) .95 – 1.0 | .98 (.02) .92 – 1.0 | .99 (.02) .94 – 1.0 | .98 (.02) .92 – 1.0 | .98 (.03) .89 – 1.0 |
| **BART Speed** | 637.83 (429.65) 0.0 – 1702.88 | 682.47 (389.64) 0.0 – 1691.61 | 629.39 (372.43) 154.3 – 1377.13 | 718.27 (306.3) 147.18 – 1538.46 | 631.53 (335.99) 0.0 – 1473.3 |
| **BART Risk** | .72 (.11) .43 – .93 | .70 (.12) .39 – .90 | .63 (.1) .44 – .78 | .65 (.10) .39 – .82 | .62 (.11) .41 – .84 |
| **PVT Speed** | 5.25 (.24) 4.75 – 5.73 | 5.38 (.25) 5.02 – 5.89 | 5.30 (.34) 4.82 – 6.22 | 5.34 (.34) 4.81 – 6.28 | 5.34 (.41) 4.66 – 6.34 |
| **PVT Accuracy** | .97 (.04) .81 – 1.0 | .96 (.03) .91 – 1.0 | .97 (.02) .91 – 1.0 | .96 (.05) .82 – 1.0 | .96 (.03) .87 – 1.0 |
| **MPT Speed** | 1061.18 (153.53) 855.5 – 1434.3 | 1083.93 (140.01) 844.1 – 1444.7 | 1056.56 (126.8) 821.7 – 1354.7 | 1078.51 (177.39) 858.1 – 1655.0 | 996.36 (129.77) 777.5 – 1283.2 |
