# Supplementary material for: Cognitive performance in ISS astronauts on 6-month low earth orbit missions
Source: Front Physiol. 2024 Nov 20;15:1451269. doi: 10.3389/fphys.2024.1451269 (PMC11614644; doi:10.3389/fphys.2024.1451269)
Supplement: Supplementary file 2 [file Image1.pdf]

Supplemental Figure 1. Uncorrected Speed and Accuracy Raw Scores Over Mission Phase

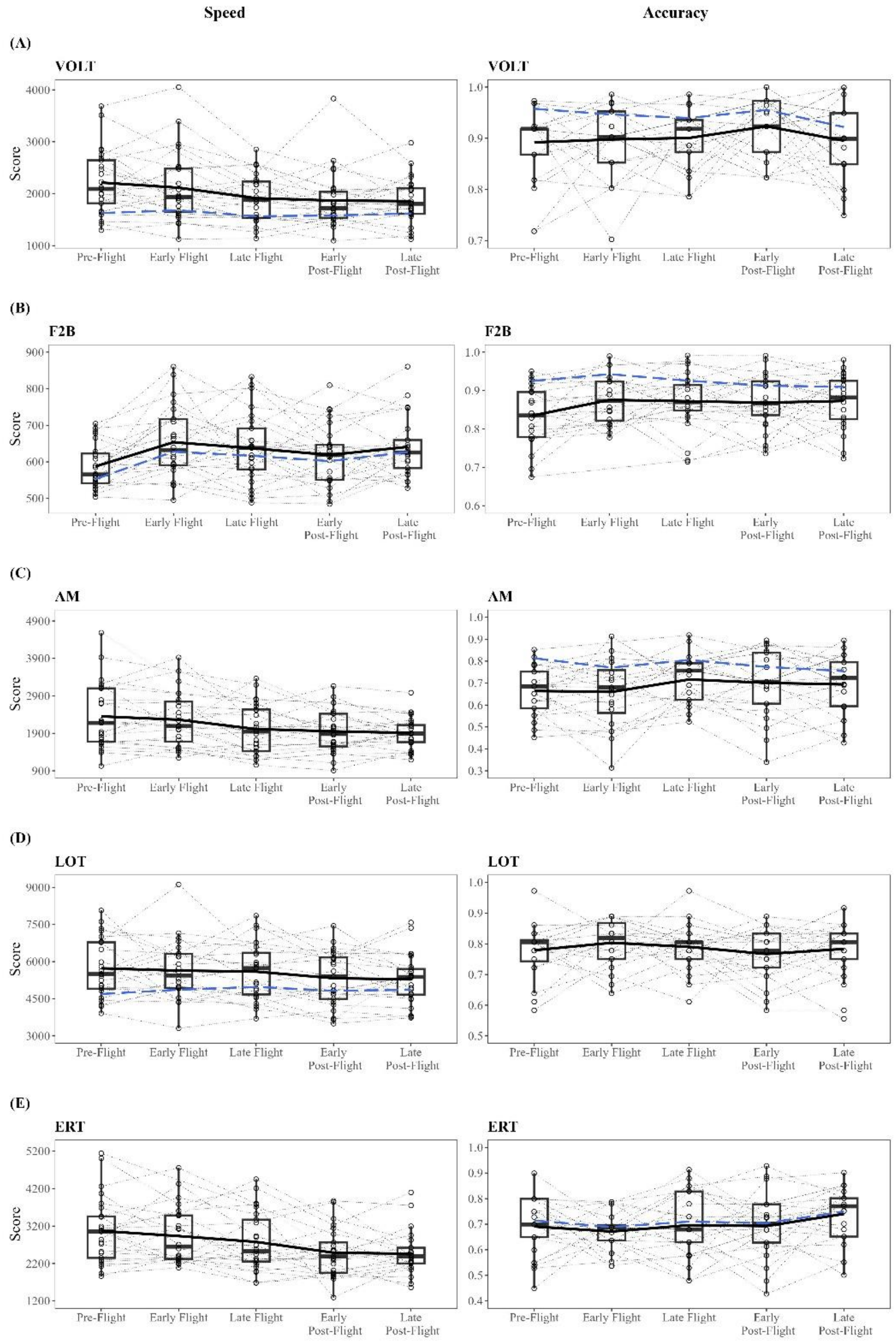

Note: Raw speed scores are represented in in milliseconds and higher values indicate slower performance. Raw accuracy scores range from 0 to 1 and higher scores indicate more accuracy performance. Blue dashed line represents the correction curve. VOLT = Visual Object Learning Test (A); F2B = Fractal 2-Back (B); AM = Abstract Matching Test (C); LOT = Line Orientation Test (D); ERT = Emotion Recognition Test (ERT).

Supplemental Figure 2. Uncorrected Speed and Accuracy Raw Scores Over Mission Phase Continued

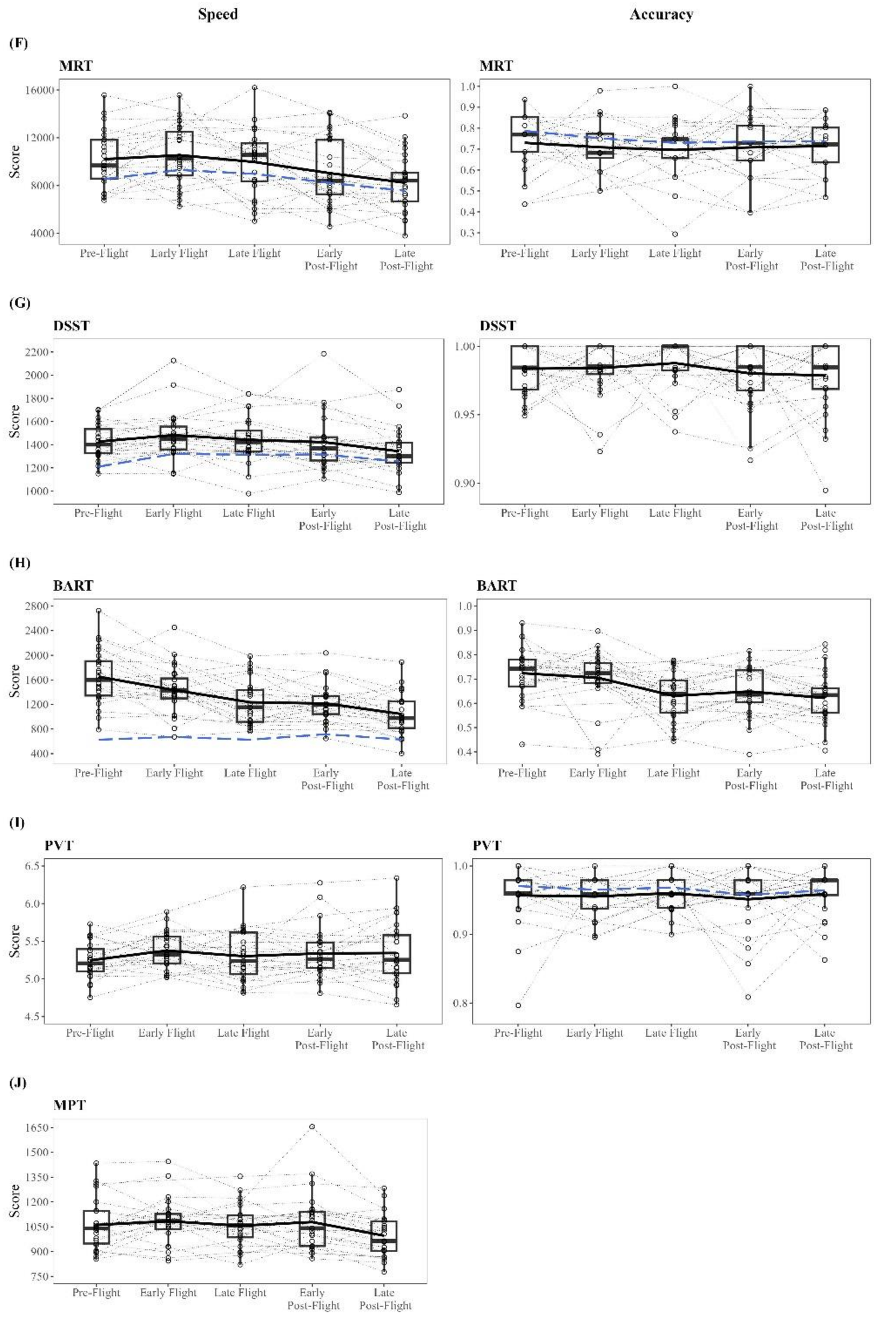

Note: Raw speed scores are represented in milliseconds and higher values indicate slower performance. Raw accuracy scores range from 0 to 1 and higher scores indicate more accuracy performance. Blue dashed line represents the correction curve. MRT = Matrix Reasoning Test (F); DSST = Digit Symbol Substitution Task (G); BART = Balloon Analog Risk Test (H); PVT = Psychomotor Vigilance Task (I); MPT = Motor Praxis Task (J).
